# The effect of radiofrequency ablation on pain score and non-steroidal painkiller use in osteoid osteoma patients

**DOI:** 10.1186/s12880-023-01113-3

**Published:** 2023-10-18

**Authors:** Abdullah Soydan Mahmutoğlu, Fatma Zeynep Arslan, Mehmet Karagülle, Mehmet Semih Çakır, Özdeş Mahmutoğlu

**Affiliations:** 1 Department of Radiology, İstanbul Training and Research Hospital, İstanbul, Turkey; 2 Department of Radiology, Basaksehir Cam and Sakura City Hospital, İstanbul, Turkey; 3 Department of Radiology, Şişli Hamidiye Etfal Training and Research Hospital, İstanbul, Turkey

## Abstract

**Aim:**

CT-guided radiofrequency ablation (RFA) is among the thermal ablative procedures and provides great benefits with a minimally invasive procedure. In this prospective study, we aimed to reveal the significance of a multidisciplinary method in reducing the recurrence and complications in osteoid osteoma patients with CT-guided RFA performed by a team of experts in the field.

**Materials and methods:**

A total of consecutive 40 patients with osteoid osteoma were prospectively evaluated and treated with CT-guided RFA. Before and the post ablation the visual analog scale (VAS) and use of nonsteroidal anti-inflammatory drugs (NSAIDS) were compared.

**Results:**

Post-ablation VAS of the patients at the 1st week and 3rd month after the procedure decreased significantly (p < 0.01) compared to the pre-ablation. The frequency of NSAID use after the ablation decreased significantly (p < 0.01) compared to the pre-ablation time. The pre-procedure NSAID use of our patients included in the study was average 6.93 per week, the NSAID use in the 3rd month post-procedure controls was average 0.53 per week. Recurrence was detected in 4 of our patients, 36 patients had complete recovery.

**Conclusion:**

Radiofrequency ablation is an effective treatment method in the management of osteoid osteomas. Radiofrequency ablation has low recurrence rates and provides rapid regression in patients’ pain after treatment.

## Introduction

Osteoid osteoma (OO) is a small, well-circumscribed, with a nidus surrounding sclerotic bone, non-progressive, benign osteoblastic lesion that can cause severe pain disproportionate to its size. OO is the third most common benign bone lesion [1]. OO has a typical image on computed tomography (CT), and it has been reported that CT sensitivity specificity is higher compared to magnetic resonance imaging (MRI) [2, 3]. In MRI, it has been reported that dynamic series are superior to CT for diagnosis in OOs that do not show typical localizations, especially by rapidly recognizing nidus in atypical localizations [2].

CT-guided radiofrequency ablation (RFA) is among the thermal ablative procedures and provides quick and reliable imaging. It provides great benefits with a minimally invasive procedure in locations that are not easily accessible, such as the femoral neck or intraarticular [4]. In CT-guided RFA, the recurrence rate is 5%, but the postoperative complication rate is much lower compared to surgery and the success rate is high [5, 6]. As the high frequency alternating current passes through the tissues, it loses energy by radiating heat, and this thermal energy causes ablation and necrosis within the tissues. Since there is no simultaneous visualization for complete removal of the nidus during surgery, a significant amount of bone tissue can be removed, which may cause bone weakness and the need for bone grafting. Despite all this effort, surgical recurrence rates are not very low due to the difficulties in the complete removal of the nidus, and it has been reported in recent studies that the recurrence rate after surgery ranges from 4.5 to 25% [5].

In this prospective study, we aimed to reveal the significance of a multidisciplinary method in reducing the recurrence and complications in OO patients with CT-guided RFA performed by a team of experts in the field.

## Patients and methods

### General data

A total of consecutive 40 patients with OO were prospectively evaluated and treated with CT-guided RFA between the years of 2019–2022. Patients were assessed on multimodality images (x-ray, CT and MRI). The procedures were performed by an interventional radiologist with 7-years of experienced in ablation and interventional procedures(M.K.).

Patients whose nidus can be seen on CT, lesions which are not located superficially close to the skin, are not located very close to nerve roots and important vascular structures, patients who can receive anesthesia, do not have active infection in the area to be entered percutaneously for treatment, meet the criteria of INR < 1.3, platelet count < 50,000, and have not hemophilia, who did not have diseases that would cause bleeding diathesis were included in our study. Lesions larger than 15 mm, pregnant patients and patients with fractures were excluded from our study. Three patients who did not meet our inclusion criteria were excluded from our study and referred for surgery.

In the detailed anamnesis of the patients, the number of times a week they used nonsteroidal anti-inflammatory drugs (NSAIDS) and the scores they gave to their pain according to the visual analog scale (VAS) were noted. After the procedure, the average number of times a week they used NSAIDs and the scores they gave for their pain in the first week and third month were noted according to the typical numerical pain scale of the VAS. The VAS was used to evaluate pain scoring [7]. Numbers 0–10 were written on the horizontal line. 0 is account for no pain, 10 is account for excruciating pain. For the value to be measured, the patient was asked to mark the point felt and thought. According to this system, a pain score of 7 and above corresponded to severe pain.

### RFA ablation

The procedures were performed under general and local anesthesia. Sedoanalgesia was applied to these patients. For this purpose, midazolam 0.05–0.1 mg/kg, Fentanyl 1mcg/kg and propofol 1 mg/kg were administered. Patients were monitored with VAS during the procedure. After the suitable anesthesia performed, CT scan mapping is engaged. The skin was cleaned with a disinfectant, local anesthetic agent (10 cc) injected and an incision was made into the skin. In 3 of our patients, the bone tissue was penetrated with a drill and the RFA probe was advanced. (18 F; APRO Korea Inc., Gunpo, Korea). The bone cortex was passed with the hammer in the rest of patients [8]. A bone penetration needle (11 F; APRO Korea Inc., Gunpo, Korea) is then inserted into the lesion nidus. The bone needle inner stylet is removed, leaving the outer layer. RFA needle is sent through the outer layer and fixed in the lesion nidus. Afterwards, the burning process is applied at 100 degrees for 4–6 min. Then the process is terminated.

### CT and MRI technique

A CT device (32-slice scanner) is used for the evaluation. MRI was performed using 1.5-T MRI scanner [Magnetom Aera; Siemens Healthcare, Germany] and various dedicated coils depending on the examined region (e.g. knee coil, flex coil, body coil). T1-weighted (T1W), T2W sequence, Short-Tau-Inversion-Recovery (STIR) sequence T1W sequence with fat saturation on axial and sagittal planes after and before the contrast media administration were performed.

### Statistical analysis

Data obtained from personal information forms and scales were transferred to a computer by SPSS (Statistical Package Programme for Social Sciences 22.0) program, and the data were analyzed by this program. The continuous variables (age, nidus diameters, periosteal reaction, pre-op pain score, post-op pain at 1st week, post-op pain at 3rd month, pre-op and post-op NSAID usage) were presented as arithmetic mean ± standard deviation, while quantitative data (gender, location of lesions, nidus locasion, nidus contrast enhancement, and precences of recurrence or residue) were presented as number and percentages (%). The distribution of variables is measured with the Kolmogorov-Smirnov test. In order to compare the quantitative variables of pain scores and NSAID usage (pre-op pain score, post-op pain at 1st week, post-op pain at 3rd month, pre-op and post-op NSAID usage), the Wilcoxon test was used for these data that were not normally distributed. The p-value was accepted < 0.05 at a 95% confidence interval.

### Clinical responce

When evaluating the response to treatment, we primarily considered the patient’s clinic and NSAID use status. We categorized patients into four subgroup according to treatment response.

Clinical complete response: Patients who defined their pain score as 0 at the 3rd month after the procedure and who did not use any NSAID medication were considered as.

Clinical partial response: A minimum 2-point decrease in post-procedure pain score compared to preprocedure or a decrease in NSAID use by more than 25%.

Recurrence: Patients whose VAS and NSAID use decreased at the 1st week follow-up after the procedure, but who did not meet the partial response criteria for VAS and NSAID use at the 3rd month follow-up.

No clinical response to treatment: Patients whose VAS and NSAID use increased at follow-ups after the procedure.

Additionally, patients were scheduled for a 3-month follow-up MRI examination. However, we could not perform MRI for many of our patients.We based treatment evaluation on clinical response.In patients for whom we can perform MRI; In T2W images, regression of edema, necrosis after ablation, and disappearance of the nidus were seen in our patients in whom we demonsrated a complete clinical response.

## Results

Forty OOs were included in our study. The mean age of our patients was 18 and 13 of the patients participating in the study were women. Most of the lesions were located in the femur neck (n:12, 30%). Eleven lesions (27.5%) were located in the femur diaphysis and 1 lesion (2.5%) was intraarticular. Locations of other lesions are shown in Table 1.

**Fig. 1:**
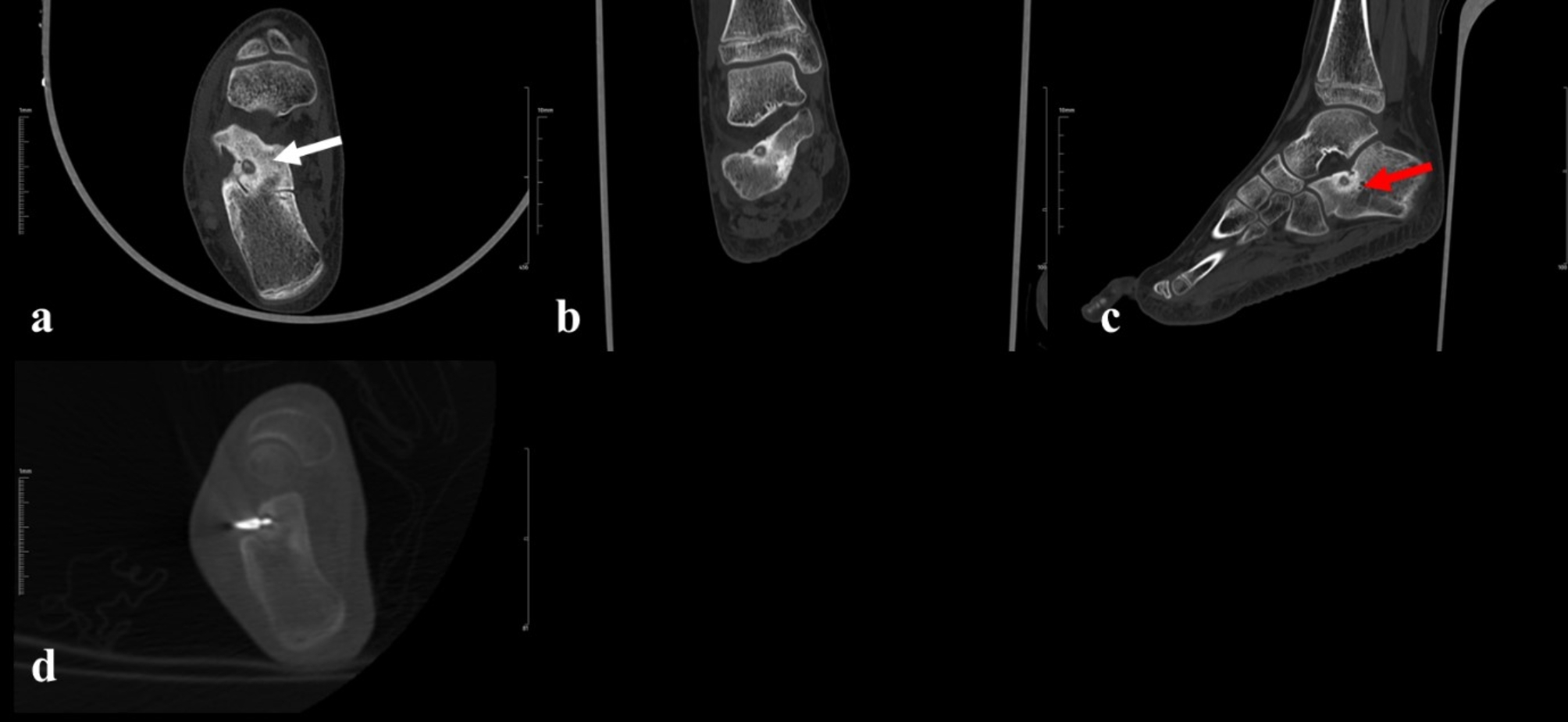
In an 11-year-old female patient admitted to outpatient clinic with feet pain, peripherally sclerotic and centrally hypodense lesion located in the calcaneus is shown on CT images (red arrow). **a**.) in pre-op axial sections, **b**.) in pre-op coronal sections, **c**.) in pre-op sagittal sections the nidus diameter was 7 mm, and the periosteal reaction (White arrow) thickness was 4 mm, **d**.) and in the image during processing a bone penetration needle (11 F; APRO Korea Inc., Gunpo, Korea) is inserted into the lesion nidus. Afterwards, the burning process is applied at 100 degrees for 4–6 min. The pre-procedural pain score was 10, the pain score decreased to 1 point in the 1st week and 3rd month after the procedure. The patient uses NSAIDs 7 times a week (1 daily) before the procedure, she does not use any NSAIDs after the procedure

**Fig. 2:**
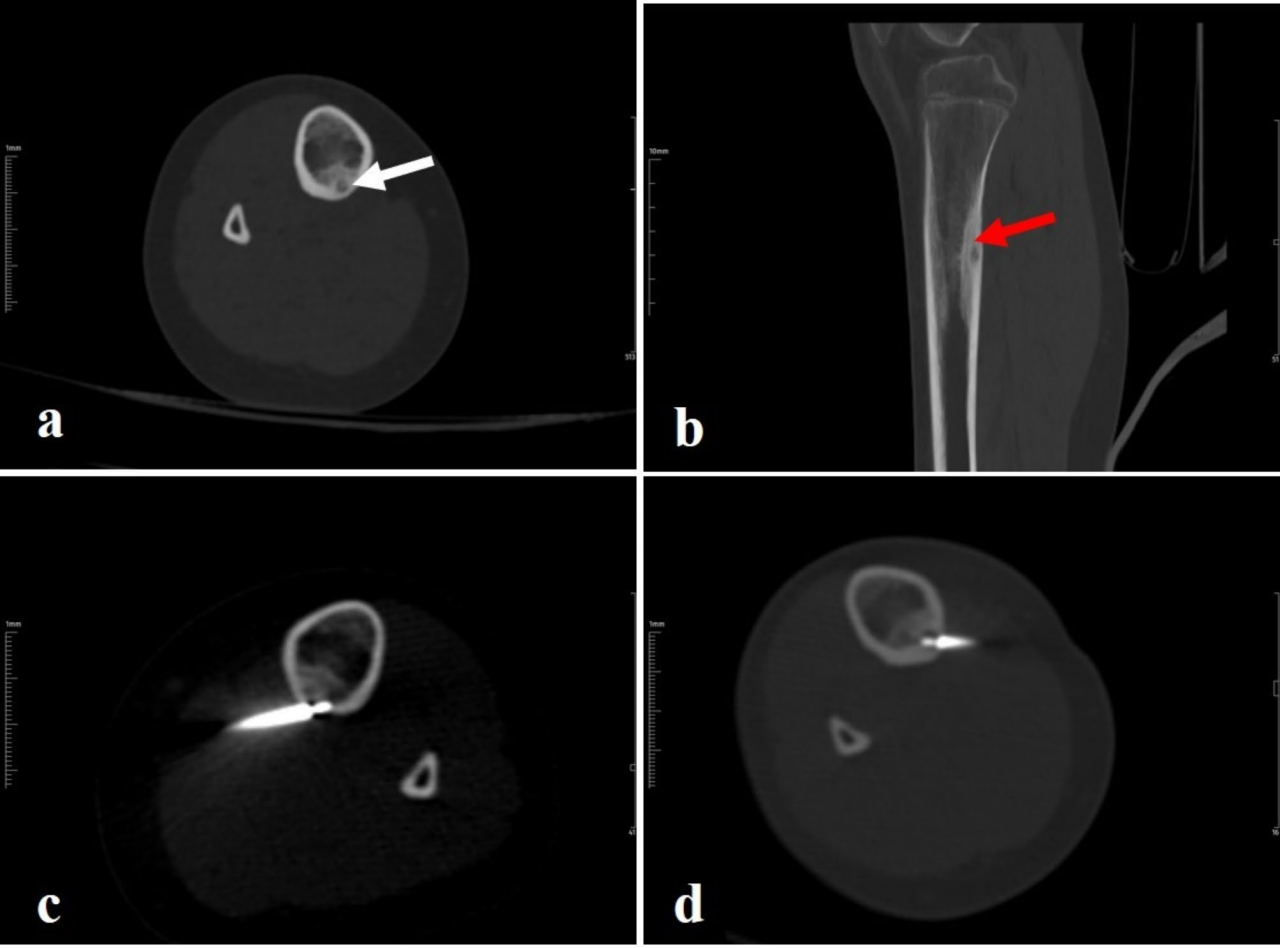
In an 9-year-old female patient recurrence is seen after the RFA. **a**.) on axial images, **b**.) on coronal images the lesion located in the cortex of the proximal tibia is seen on CT images. The lesion is peripherally sclerotic and centrally hypodense. the nidus (white arrow) diameter was 4.7 mm, and the periosteal reaction (red arrow) thickness was 4.5 mm, **c**.) the image is shown first RFA, **d**.) the image is shown second RFA, after the first ablation. The pain score was 9 before the first ablation, the pain score after RFA was 3 points at the 1st week and 4 points at the 3rd month. The patient was using NSAIDs 7 times a week before the first procedure, she started taking NSAIDs 4 times a week after the first ablation. Our patient was considered to have relapse because the patients’ pain and NSAID use did not improve effectively. A second RFA was performed 6 months after the first ablation. Before the second RFA, the patient had a pain score of 7 and was taking NSAIDs 4 times a week. After the second RFA, the pain score decreased to 0 points in the 1st week and 3rd month. NSAID use was also 0

**Fig. 3:**
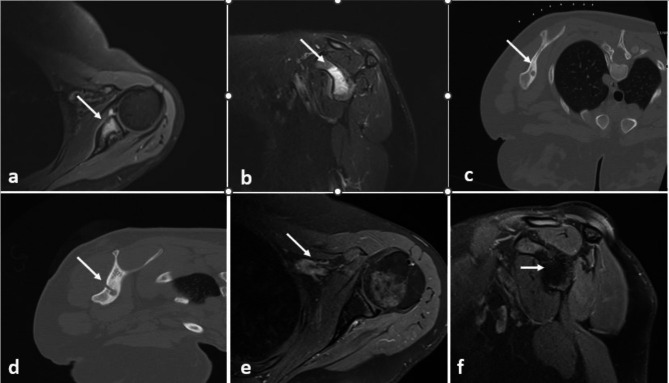
Pre-op axial fat-suppressed T2W **a**). axial and **b**). sagittal images, **c**). pre-op CT axial images, **d**). post-op fat-suppressed T2W axial images, 3rd month post-op fat surpressed T2W **e**). axial and **f**). sagittal images are shown. In the pre-op images, edema is observed within the osteoid osteoma nidus at the level of the coracoid process. In the post-ablation images, it is seen that the edema within the lesion has dissolved, gradually turned into post ablation necrosis and the nidus has disappeared.

All OOs showed cortical localization. Mean diameter of the nidus was 6.40 mm (min- max: 2.6 12.0 mm). Average 4.84 mm (min-max: 0-10.5 mm) thickness periosteal reaction was observed. The pre-procedure mean VAS pain score is 8 points. The mean VAS pain score was 0.53 points at the first week after the procedure. The 3rd month post-procedure mean VAS pain score was 0.53 points. Post-procedure pain scores at the 1st week and 3rd month after the procedure decreased significantly (p < 0.01) compared to the pre-procedure. The frequency of NSAID use after the procedure decreased significantly (p < 0.01) compared to the pre-procedure (Table 2; Figs. 1 and 2). The pre-procedure NSAID use of our patients included in the study was average 6.93 per week, the NSAID use in the 3rd month post-procedure controls was average 0.53 per week. Recurrence was detected in 4 of our patients, 36 patients had complete recovery. In our study, there were no patients with clinical partial response or no clinical response to ablation (Fig. 3). There were no major or minor complications of RF ablation in the patients in our study.

## Discussion

RFA is accepted as the gold standard because it creates a quick and practical treatment opportunity for OO. And for this reason, there are many studies investigating the success of OO treatment [9]. The complete disappearance of pain for 2 years in patients undergoing RFA is considered a cure [10]. And this success rate reaches almost 95% [9]. However, residual pain is not always due to unsuccessful nidus ablation, it can also be seen due to incorrect position of the needle, damage of adjacent soft tissues [9, 11]. Unfortunately, in large lesions, the needle must be positioned more than once and interventions at different angles are required. In our study, the nidus diameter was 10 mm in our patient who had significant pain after the procedure. Recurrence was detected in other patients who had pain after the procedure. Vanderschueren et al. [12] found that lower age can prodispose a risk for lower recurrence. Baal et al. [13] reported that recurrence is more frequently seen in patients with younger age, females, broad lesions and located in eccentric places. And they found combination of conditions at highest risk for recurrence is female patient and the eccentric locations. Differently from this, half of the patients with recurrence in our patients were women, and recurrence was observed in typical localizations. The fact that the recurrence rate was much lower in our study reveals that perhaps one of the most important reasons for recurrence is operator experience and manual dexterity. In our study, unlike previous studies, it was observed that there was no significant relationship between recurrence and age, gender, location and many factors. However, although we commented that the difference in recurrence rates between studies was due to operator dependency, interobserver variability was not examined in our study. This still remains an unanswered research issue.

Osteoblastomas tend to be larger in size compared to OOs and involve atypical localizations such as the axial skeleton [14]. Gümüştaş et al. [15] reported that the most important problem in the management of OOs located in abnormal locations is establish an appropriate diagnosis. Perhaps one of the reasons why recurrence was more common in atypically located; small osteoblastomas were mistaken for OO. In atypical locations, it may be difficult to reach the nidus because of the excessive thickening of the cortex as a result of the periosteal reaction and the formation of sclerosis in the medullary canal [9]. In such cases with excessive thickening of the cortex, it is impossible to reach the nidus by hammering, but this number is fortunately reltively small. In our study, hammering was not sufficient in only 4 patients and drilling with a battery-powered tool was used. We mostly prefer hammering since there are no danger for the thermal damage and soft tissue invagination.

RFA is a nearly perfect minimally invasive method for OO, it has some potential complications. These complications were much less frequent compared to surgery, and it was reported as 0.9% in a large series of 557 patients treated [16]. The skin burn, neural injury, bleeding and infection are among these reported complications. Neural injury can be seen more often as a complication in spinal and metacarpal localized OOs. In order to prevent neural injury, it is recommended to place the electrodes at least 1 cm away from the main neural networks [10].Oc et al. [17] conducted a study showing the major and minor complications of RFA. They reported that complications occurred mostly in lesions located on the tibia.These complications were second-degree burns, superficial skin infection, the probe tip was broken and remained within the bone, intramuscular hematoma and numbness in the fingers developed in a lesion located in the metacarpus.

There is no detemined follow-up program after RFA treatment. however, we called our patients for check-ups in the 1st week and 3rd month after the procedure and followed them for 1 year. In the follow-ups, the decrease in size or disappearance of the nidus is examined with CT control.Erbaş et reported in a study there is no significant difference on imaging findings between follow-up periods. And they argued that single dynamic contrast-enhanced MRI should be taken within the first 3 months after treatment in the group of patients whose pain completely disappeared during follow-ups due to lack of ionizing radiation. No algorithm has been established for the follow-up intervals after RFA treatment, and an ablation technique that must be strictly followed has not been determined. Some researchers recommend direct overheating at 90 °C by passing the initial phase in which the electrodes are heated, and then lowering the temperature and recommending 2 min plateau at 60 °C. Thus, the total intervention time will be over 15 min [18, 19]. Another treatment algorithm was 90 °C RFA for 2 min. The success of each of these techniques was over 90% [20].In our study, the burning process is applied at 100 degrees for 4–6 min.

59% of the authors did not use any post-procedure analgesic while the remainder prescribe oral analgesic medication to their patients after the procedure [21].Gülenç et al. [22] found in their sudy, the mean duration of pain was 9.9 days, which was a short time period. They reported that the most important reason for the duration of the pain to be so short is to perform the interventional procedure with appropriate technique and manual dexterity. In our study, post-ablation VAS of the patients at the 1st week and 3rd month after the procedure decreased significantly (p < 0.05) compared to the pre-ablation. The frequency of NSAID use after the ablation decreased significantly (p < 0.05) compared to the pre-ablation time. Lesions with atypical clinical findings, localization, or imaging findings require biopsy to exclude malignant tumors such as osteoblastoma. In addition, Brodie’s abscess, some fracture and hemangioma can mimic OO [22].Laredo et al. [23]. conducted a study using two different types of needles (11, 12.5 and 14 gauge cannula) and the success rate of biopsy is varying between 66.1 and 81.4%. However, they reported that a significant number of biopsies resulted in nondiagnostic results regardless of needle and operator technique.

Rosenthal et al. [24] reported that 27% of their results were nondiagnostic. Soliman et al. [25] found that 48% of patients were non diagnostic. However, the lesions of these patients with unsatisfactory results were treated with RFA, suggesting that non-diagnostic biopsies are most likely to be OO [23]. Another view is that the reason for the negative biopsy result is insufficient material to be obtained or inappropriate pathological sections taken. In studies, it has been reported that the success of treatment is similar and at a high rate in patients with nondiagnostic biopsy results [22].

There were some limitations in our study. The first was relatively small sample size. Another limitation was that interobserver variability was not examined. In addition, biopsy was not taken from the lesions before the procedure, which was the main limitation of our study, however, most of our patients were cured. Future studies examining large sample sizes and evaluating interobserver variability will contribute to the literature. In addition, it may be beneficial to conduct new studies that include MRI, which is superior to CT in dynamic series.

In conclusion: RFA is an effective treatment method that provides a high rate of treatment success with minimal procedures in the treatment of OO. And the recurrence rate is low. With RFA treatment, the patient is discharged quickly and there is a significant regression in pain.

**Table 1:** General Data of the Participants

		Min-Max			Median	Mean ± sd		
**Age**		7.0	-	44.0	17.0	18.03	±	7.23
**Gender**	Male					27		67.5%
	Female					13		32.5%
***Location***								
Femur neck						12		30.0%
Femur diaphyseal						11		27.5%
Tibia diaphyseal						5		12.5%
Femur trochanter major						3		7.5%
Calcaneus						3		7.5%
Head of Humerus (Intra-articular)						1		2.5%
Third Metacarpal						1		2.5%
Femur distal metaphysis						1		2.5%
Femur metaphyseal						1		2.5%
Tibia diaphysis						1		2.5%
Tibia metaphyseal						1		2.5%
**Nidus diameter** (mm)		2.6	-	12.0	6.0	6.40	±	1.93
**Nidus location**	cortical					40		100.0%
**Nidus contrast enhancement**	periferally					39		97.5%
	absent					1		2.5%
**Periosteal reaction**		0.0	-	10.5	4.0	4.84	±	2.54
**Pre-op pain score**		5.0	-	10.0	8.0	8.00	±	1.09
**Pre-op NSAID**		0.0	-	18.0	7.0	6.93	±	3.63
**Recurrence or residue**	yes					4		10.0%
	no					36		90.0%

***NSAID***: nonsteroidal anti-inflammatory drugs

**Table 2:** The alteration of the pain score and NSAID need

		Min-Max			Median	Mean ± sd			p	
***Pain score***										
Pre-op		5.0	-	10.0	8.0	8.00	±	1.09		
Post-op (1st week)		0.0	-	7.0	0.0	0.53	±	1.52	p < 0.001*	^w^
Post-op (3rd month)		0.0	-	5.0	0.0	0.53	±	1.45	p < 0.001*	^w^
***NSAID***										
Pre-op		0.0	-	18.0	7.0	6.93	±	3.63		
Post-op		0.0	-	7.0	0.0	0.53	±	1.63	p < 0.001*	^w^

***NSAID***: nonsteroidal anti-inflammatory drugs; ^*^: Wilcoxon test

## Data Availability

The datasets generated and/or analysed during the current study are not publicly available due [The consent from all patients to publish this data did not obtain] but are available from the corresponding author on reasonable request.
